# Sleep duration differences between children of migrant and native origins

**DOI:** 10.1007/s10389-015-0665-8

**Published:** 2015-04-03

**Authors:** L. J. W. (Wim) Labree, H. (Dike) van de Mheen, F. F. H. (Frans) Rutten, G. (Gerda) Rodenburg, G. T. (Gerrit) Koopmans, M. (Marleen) Foets

**Affiliations:** 1 Institute of Health Policy and Management, Erasmus University, P.O. Box 1738, 3000 DR Rotterdam, The Netherlands; 2 IVO Addiction Research Institute, Heemraadssingel 194, 3021 DM Rotterdam, The Netherlands; 3 Erasmus MC, University Medical Center, P.O. Box 2040, 3000 CA Rotterdam, The Netherlands; 4 Department of Health Promotion, Maastricht University, P.O. Box 616, 6200 MD Maastricht, The Netherlands

**Keywords:** Child health, Transients and migrants, Sleep, Parenting, The Netherlands

## Abstract

**Aim:**

To explore whether primary school children of migrant and native Dutch origins differ regarding their sleep duration per night, a risk for overweight and obesity, and to determine to what degree differences in parenting styles contribute to these differences.

**Subjects and methods:**

A cross-sectional survey, including 1,943 children aged 8-9 years old and their primary caregivers, was performed. Data were collected from primary schools in cities and adjacent municipalities in The Netherlands: Eindhoven and Rotterdam. The outcome measure was mean sleep duration per night. The main independent variable was migrant background, based on the country of birth of the parents. A possible mediating variable was parenting style (rejecting, neglecting, permissive, authoritarian, authoritative). Age and sex of the child as well as parental socioeconomic status, as indicated by educational level, were added as confounders.

**Results:**

Dutch children have the highest sleep duration: more than 11 h (mean = 670.1; SD = 27.7). All migrant children show less than 11 h of sleep per night. Migrant children of non-Western origin, especially Turkish and Moroccan children, show the lowest sleep duration per night. Parenting styles do not contribute to these differences.

**Conclusion:**

Migrant background is associated with sleep duration. As children of migrant origin are, in general, at higher risk for overweight and obesity and sleep duration is regarded as a risk factor for overweight and obesity, further investigation of this association is needed.

## Introduction

All over the world, the overweight and obesity epidemic has become an enormous threat for public health as a consequence of the severe impact on quality of life, morbidity and even mortality (Aranceta et al. 2009; Nguyen and El-Serag 2010). Both obese adults and obese children may experience such negative consequences in the short or long run (Reilly and Kelly 2005).

In the European region, the prevalence rates of overweight and obesity are still growing rapidly, especially in young people (Berghöfer et al. 2008). Childhood overweight may develop over time into adolescent obesity (Reilly et al. 2011). Also, parental overweight is regarded as an important risk factor predicting weight problems of their children (Murrin et al. 2012).

In spite of multiple risk factors, the increase of childhood overweight and obesity is generally the result of energy balance disorders. Factors that affect childhood overweight and obesity include lifestyle behaviors, which in turn are shaped by parenting practices. These practices are also influenced by child characteristics, such as age and gender, which are also risk factors for overweight and obesity (Davison and Birch 2001). In addition, according to a systematic review of the European literature, migration seems to play a role, as migrant children are even more at risk of overweight and obesity than their indigenous counterparts (Labree et al. 2011). Indeed, immigration to high-income European countries may alter lifestyles, such as alterations in physical activity and dietary intake (Méjean et al. 2009; Delavari et al. 2013).

Apart from the more traditional risk factors of overweight and obesity, short sleep duration is regarded as an independent risk factor for overweight and obesity (Beccuti and Pannain 2011; Knutson 2012) in both adulthood and childhood (Liu et al. 2012; Rutters et al. 2010). This association can be explained by the fact that, in order to counterbalance the additional energy expenditure resulting from increased time awake, a higher food intake than needed is consumed by the human body (Hasler et al. 2004). Another explanation for this association is that short sleep duration may disrupt the hormones regulating appetite, which may increase the appetite for carbohydrate-rich foods (Spiegel et al. 2004). Finally, sleep deprivation is associated with decreased glucose tolerance, which is also regarded as a potential risk for overweight and obesity (Van Cauter et al. 2008).

Short sleep duration can be due to several factors. Multiple persons sleeping in one room, the presence of daylight and room temperature negatively affect sleep duration (Magee et al. 2010). Additionally, there is evidence in the literature of an association between children with socially disadvantaged backgrounds and low levels of sleep duration (Magee et al. 2010; O’Dea et al. 2012). Furthermore, ethnicity seems to play a role. Wong and colleagues (2013) found that primary school children from underserved minorities, living in the USA and belonging to ethnic minority groups, showed an increased overweight and obesity risk when the suggested guidelines from the National Sleep Foundation were not followed. These guidelines recommend a sleep duration of 10-11 h per night for these children.

In Europe, differences in sleep duration between specific migrant groups, in both adults and children, have, to our knowledge, not been studied. Given the fact that overweight and obesity are more prevalent among migrant children as compared to the native population, we hypothesize that migrant children have less sleep than non-migrant children.

Furthermore, parents have a key role in stimulating optimal sleep duration (de Jong et al. 2012). Therefore, it is hypothesized that parenting styles, showing more behavioral control, influence the child’s sleep in a positive way, as this type of control reflects the regulation of the child’s behavior through firm and consistent discipline by strict house rules.

## Methods

### Study aim

**Fig. 1:**
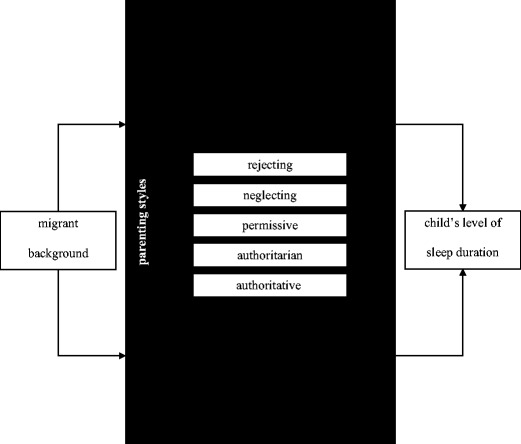
Conceptual model of the influence of the home environment on sleep duration

The main aim of this study is to compare the sleep duration per night between migrant and native primary school children in The Netherlands. An additional study aim is to determine whether differences between these children can be explained by parenting styles of the primary caregivers, taking into account the parental socioeconomic position and age and sex of the children. The conceptual model is presented in Fig. 1.

### Background study

In the context of the IVO Nutrition and Physical Activity Child CohorT (INPACT) study, a cross-sectional study was performed. This study is a collaborative project between the Institute of Health Policy and Management, a department of the Erasmus University Rotterdam, and the IVO Addiction Research Institute.

In this 4-year longitudinal project, factors in the obesogenic environment within the developmental trajectory of weight are studied. Subjects are 8–9-year-old Dutch primary school children. For the present article, data collected at baseline were analyzed (2009).

### Study population

Our study population consists of parent-child dyads. These dyads were recruited from primary schools in two large cities in The Netherlands and adjacent municipalities around these cities: Eindhoven and Rotterdam. Eindhoven is the fifth largest city, and Rotterdam is the second. Children from different migrant backgrounds, Western descent or non-Western descent, attend school in these cities.

In this study, 1,943 dyads (61.5 %) decided to participate. Schools decided to participate in the INPACT study based on a letter in which its aim and relevance were explained.

Schools were excluded if they were participating in any prevention programs because these were expected to influence our measures. Participation in this study was on the basis of informed consent of the children’s primary caregivers.

The study was approved by the medical ethics committee of the Erasmus MC, University Medical Center Rotterdam.

### Measurements

The general part of the questionnaire included questions on the age and sex of the children and the socioeconomic position and migrant background of the parents.

Socioeconomic status was assessed by the highest educational level achieved by one of the parents, divided into three categories: low (primary school, lower vocational or general education), middle (secondary school, intermediate vocational school) and high (higher vocational school or university). Children were considered as having a migrant background when at least one of their parents was born outside The Netherlands, according to current Dutch practice (CBS 2000). Also, based on this classification, five groups in our sample were distinguished: three homogeneous groups, consisting of native Dutch children (*n* = 1,546), children with a Turkish background (*n* = 93) and children with a Moroccan background (*n* = 66). Also, two supplementary groups were included containing more heterogeneous groups of children. In these groups, we distinguished children from a variety of non-Western countries, other than Turkey and Morocco, labeled ‘other non-Western children’ (*n* = 105), and from a variety of Western countries other than The Netherlands, labeled ‘other Western children’ (*n* = 133).

#### Outcome measures: sleep duration

Sleep duration was measured by two questions. The primary caregiver had to report the time, based on an average school day, their children went to sleep and awoke. The answering scales for these questions consisted of several time categories with half-hour intervals, of which two broader time categories concerned a specific time in the evening or in the morning (e.g., “earlier than 6 a.m.” or “later than 10:30 p.m.”).

Based on the answers, we calculated the mean number of minutes that the child sleeps per night during a normal school week. In case a primary caregiver made use of the broader time categories, we added or subtracted an extra half hour to the total sleep duration.

#### Parenting styles

The parenting style of the primary caregiver was determined based on the Dutch translation of an instrument by Steinberg and colleagues (Beyers and Goossens 1999; Lamborn et al. 1991; Steinberg et al. 1989). This instrument consists of 22 items that assess three parenting-style dimensions: support, behavioral control and psychological control. Respondents could answer on a Likert scale ranging from -2 (completely disagree) to +2 (completely agree).

Support was measured with seven items, for example: “When my child gets a low grade in school, I offer to help him/her.” At the end, these items were combined into a single variable by summing the item scores, ranging from -14 (low) to + 14 (high). Behavioral control was also measured with seven items, for example: “I know exactly what my child does in his/her free time.” A sum score was determined, also ranging from -14 (low) to + 14 (high). Psychological control was measured with eight items, for example: “I make my child feel guilty when he/she gets a low grade in school.” This sum score ranged from -16 (low) to +16 (high).

In each of the dimensions, we distinguished between two groups, based on the data distribution, as a result of a median split: low and high scores. Based on these three parenting dimensions, five parenting styles were established: rejecting (low support, low behavioral control, high psychological control), neglecting (low support, low behavioral control, low psychological control), permissive (high support, low behavioral control, low psychological control), authoritarian (low support, high behavioral control, low psychological control) and authoritative (high support, high behavioral control, low psychological control). Based on this common typology, other possible combinations were left out. As our hypothesis emphasizes the influence of behavioral control, it is hypothesized that authoritarian and authoritative parenting styles (high behavioral control) lead to longer sleep durations.

### Analysis

First, our study population characteristics (*n* = 1,943) were calculated for each of the five migrant groups. In addition, to compare the sleep duration of native Dutch and migrant children and to compare parenting styles within these groups, differences were tested with a t-test or chi-square test. Our reference group consisted of native Dutch children. Second, to explore possible correlations between age, sex, educational level of the parents and parenting styles (independent variables) and child sleep duration (dependent variable), Spearman and Pearson correlation coefficients were determined. Finally, multivariate linear regression was used to investigate whether the relation between migrant background and the child’s sleep duration could be explained by differences in parenting styles, as presented in our hypothetical model. We controlled for age, sex and socioeconomic level. Missing values were excluded from the analyses. Data were analyzed using the SPSS program (version 19.0).

## Results

Characteristics of all children in the sample (*n* = 1,943) are presented in Table 1. In the upper part of this table, the age of the children (mean and SD), percentages of boys and girls, and parental educational level in each group are presented. The educational level of the parents of the native Dutch children and non-native Western children was higher than this level in the parents of the other children (*p* < 0.05).

**Table 1:** Sample characteristics

	Dutch (*n* = 1,546)	Turkish (*n* = 93)	Moroccan (*n* = 66)	Non-Western (*n* = 105)	Western (*n* = 133)
Age, M (SD)	8.2 (0.45)	8.6 (0.67)	8.5 (0.61)	8.4 (0.63)	8.3 (0.52)
[Missing]	[4]	[1]	[1]	[3]	[2]
Boys, *n* (%)	778 (50.3)	38 (40.9)	36 (54.5)	44 (41.9)	74 (55.6)
Girls, *n* (%)	768 (49.7)	55 (59.1)	30 (45.5)	61 (58.1)	59 (44.4)
Educational level parents, %					
Low	12.5	42.4^*^	34.5^*^	26.4^*^	13.9^*^
Medium	39.7	37.6^*^	37.9^*^	35.2^*^	32.8^*^
High	47.9	20.0^*^	27.6^*^	38.5^*^	53.3^*^
[Missing]	[23]	[8]	[8]	[14]	[11]
Parenting style parents, %					
Rejecting	21.1	62.3^*^	46.3^*^	58.1^*^	32.1^*^
Neglecting	20.9	3.8^*^	9.8^*^	5.4^*^	13.2^*^
Permissive	22.6	28.3^*^	22.0^*^	13.5^*^	20.8^*^
Authoritarian	10.9	1.9^*^	4.9^*^	8.1^*^	9.4^*^
Authoritative	24.4	3.8^*^	17.1^*^	14.9^*^	24.7^*^
[Missing]	[42]	[4]	[4]	[6]	[12]
Child’s sleep duration, minutes per night					
M	670.1	645.5	645.3	654.8	657.5
(SD)	(27.7)	(35.4)^*^	(34.9)^*^	(33.2)^*^	(32.5)^*^
[Missing]	[42]	[4]	[4]	[6]	[12]

^*^ *p* < 0.05

The lower part of Table 1 shows both the parenting styles of the primary caregivers and the mean sleep duration per night of the children. Overall, migrants show different parenting styles than non-migrants. Rejecting parenting styles are less prevalent among native parents compared to migrant parents, whereas neglecting parenting styles, and also parenting styles focusing on high behavioral control (authoritative style and authoritarian style), are more prevalent among native parents compared to parents of migrant origin. Additionally, Dutch parents resemble Western parents with regard to authoritarian and authoritative styles, thus having higher scores on behavioral control compared to non-Western parents.

Furthermore, mean sleep duration, expressed in minutes per night, between the groups of children differed (*p* < 0.05). Dutch children have the highest sleep duration, more than 11 h (mean = 670.1; SD = 27.7). All migrant children showed less than 11 h of sleep per night. Especially among Turkish (mean = 645.5; SD = 35.4) and Moroccan children (mean = 645.3; SD = 34.9), the minutes of sleep per night were lower. Differences between migrant and non-migrant children varied from 24.8 min to 12.6 min.

Table 2 presents bivariate correlations (Pearson’s and Spearman’s correlation coefficients, t-test) between all independent variables and the dependent variable. Besides the age and sex of the child, as well as parental educational level, the other variables did not correlate with the child’s level of sleep duration.

**Table 2:** Bivariate correlations

Child’s sleep duration			
Age, Pearson correlation coefficient	−0.114^*^ (*p* = 0.000)		
Sex, *t* value	−2.718^*^ (*p* = 0.007)	Boys, M (SD)	665.78 (30.36)
		Girls, M (SD)	669.56 (29.67)
Educational level of parents, Spearman’s rho	0.090^*^ (*p* = 0.000)	Low, M (SD)	660.18 (31.34)
		Middle, M (SD)	668.30 (30.39)
		High, M (SD)	669.94 (29.05)
Parenting style Spearman’s rho	0.033 (*p* = 0.203)	Rejecting, M (SD)	667.80 (34.60)
		Neglecting, M (SD)	670.25 (28.08)
		Permissive, M (SD)	669.27 (28.72)
		Authoritarian, M (SD)	667.99 (28.08)
		Authoritative, M (SD)	669.29 (27.23)

^*^ *p* < 0.05

In the regression analyses, using multivariate regression techniques, we tested two models (see Table 3). The first model tested the influence of migrant background on sleep duration, adjusted for age, sex and educational level. Turkish, Moroccan and other non-Western children showed lower sleep durations than native children. This was not the case for other Western children. The total variance was 5 %.

**Table 3:** Predictors of child’s sleep duration: results of multivariate regression analyses

Variables	Model 1		Model 2	
	β	t	β	t
Age	−0.08^*^	−3.04	−0.08^*^	−2.98
Sex girl	0.08^*^	2.93	0.08^*^	2.94
Background				
Turkish	−0.07^*^	−2.02	−0.06^*^	−2.26
Moroccan	−0.12^*^	−4.59	−0.12^*^	−4.56
Non-Western	−0.09^*^	−3.60	−0.09^*^	−3.48
Western	−0.03	−0.97	−0.02	−0.93
Educational level				
Middle	0.11^*^	2.65	0.11^*^	2.63
High	0.13^*^	3.19	0.13^*^	3.16
Parenting style				
Rejecting			−0.01	−0.34
Neglecting			0.01	0.32
Authoritarian			−0.01	−0.44
Authoritative			−0.00	−0.03
Adjusted R^2^	0.05		0.05	
R^2^ change	0.05^*^		0.00	

^*^ *p* < 0.05

In the last model, parenting styles were included in the regression analysis. Our reference group consisted of parents with a permissive parenting style. Once again, age, sex, migrant background and educational level were significant variables in the model. However, the parenting styles were not, and adding them did not explain the differences in sleep duration between the migrant groups. The explained variance did not increase.

## Discussion

### Conclusions

Findings from this study show that migrant children had lower levels of sleep per night compared to their indigenous counterparts. Despite the absence of specific guidelines in The Netherlands, both children of migrant origin and children of native Dutch origin follow the guidelines from the National Sleep Foundation in the USA. These guidelines do not differentiate between specific ages and advocate a total of 10–11 h of sleep per night overall for school-age children (Wong et al. 2013).

Dutch children had the significantly highest sleep duration, more than 11 h, whereas all migrant children showed less than 11 h of sleep per night. Differences between migrant and non-migrant children varied from 24.8 min (native Dutch children compared to Moroccan children) to 12.6 min (native Dutch children compared to other Western children). Furthermore, migrants showed different parenting styles than non-migrants. Rejecting parenting styles were less prevalent among native parents, whereas neglecting, authoritarian and authoritative parenting styles were more prevalent among native parents. Parenting styles neither explained differences in sleep duration nor explained ethnic differences.

As mentioned before, sleep differences between migrants and non-migrants within European countries have, to our knowledge, not been investigated. Therefore, it is impossible to make a comparison with earlier research.

This study has some limitations. First of all, it is important to point out that our study makes use of a cross-sectional design. Therefore, it is only possible to describe differences between groups at a single point of time. Causality cannot be determined by cross-sectional studies.

Second, our questionnaire only measured sleep duration by two questions: the time children went to sleep and the time children awoke on a normal school day. Also, answering scales of both questions consisted of several time categories with half-hour intervals. We did not use exact times, nor did we use self-reports by the children. Instead, primary caregivers had to fill out the questions. Although these caregivers often know what time the child goes to bed, they may not know whether the child actually is asleep or awake: awake because of not being able to sleep or awake because of not wanting to sleep, for example, reflected in secretly reading a book or listening to music. A more objective method might have been the use of physiological measurements, such as wrist actigraphy, hip accelometry or polysomnography (Liu et al. 2012). However, the high costs of these measurements prevent them from being utilized in large-population studies.

Furthermore, it is not known, apart from the sleep duration at night, how much time the children in our population sleep in the daytime, although primary school children in The Netherlands are obliged to go to school on such an average day. Furthermore, contrary to the information with regard to sleep during the week, we have no information about the sleep duration of these children during the weekend. As a result, our methodology only leads to an estimation of sleep duration based on a normal school day. However, a recent study concluded that weekday sleep duration is more strongly associated with body composition in European school children than weekend day sleep duration (Altenburg et al. 2013).

Apart from sleep duration, it would be interesting to take into account differences in sleep quality, as both duration and quality may affect the development of overweight and obesity in children (Bawazeer et al. 2009). However, no questions with regard to sleep quality were included in the questionnaire.

The utility of country of birth of the parents, in order to classify migrants in our sample, has the advantage of being objective and stable (Stronks et al. 2009). The classification of other non-native children into heterogeneous groups, other Western and other non-Western children, was needed, despite the classification into the other two homogeneous groups, children with Turkish and Moroccan backgrounds, because of the number of children within the separate groups. Notwithstanding this heterogeneity, we found that, concerning sleep duration level, Western children resemble the native Dutch children more and the non-Western children resemble the Turkish and the Moroccan children more.

There might not be a direct relationship between parenting style and sleep duration, as behavior of children in their bedrooms is often out of sight of the parents. Perhaps features in the child’s home environment offer a better explanation, such as temperature, light or noise (Knutson 2012). Therefore, further investigation into the association between sleep duration and migrant background and insight into factors affecting the sleep duration and sleep quality are needed.

Apart from the need for research, tackling overweight and obesity is only possible by identifying specific risk factors followed by developing adequate prevention programs (Boone-Heinonen et al. 2008). Therefore, our findings might have important clinical implications for the prevention and treatment of overweight and obesity in children, because sleep duration is a risk factor for overweight and obesity that is regarded as potentially modifiable as it is relatively easy for parents to control in their homes (Taheri 2006). However, sleep duration is not often included in policy and environmental strategies (Brennan et al., 2014). We emphasize the importance of sleep duration in prevention activities.

In general, it is advised that interventions should pay attention to cultural aspects of the targeted population (Kocken et al., 2012). The results of this study, distinguishing migrants from non-migrants, do support this advice. Also, current prevention programs can be more effective if preventive interventions are aimed to improve the community (Moreno et al., 2013), for example, regarding migrant families who often spend much time together. However, none of the common interventions seem to distinguish migrant from non-migrant children.

According to this study, differences do exist between these children with regard to sleep duration. Additionally, the prevalence of overweight and obesity is significantly higher in migrant children. Our results strongly emphasize the need for more attention in research and policy to cultural and community aspects, not only to prevent overweight and obesity among children of migrant origin, but in order to decrease overweight and obesity among all children.

## Appendix

Appendix. Parenting styles: questionnaire items

**Table:**

(1) Support
• When my child gets a low grade in school, I offer to help him/her
• When my child gets a low grade in school, I encourage him/her to do better
• When my child gets a high grade in school, I show my approval and admiration
• I help my child with his/her homework when he/she does not understand
• My child can count on me when he/she has troubles
• I make time for my child
• We often engage in family activities
(2) Behavioral control
• I know exactly what my child does in his/her free time
• I know exactly what my child does after school
• I know exactly where my child goes after school
• When someone in our family leaves home, he/she tells this to the other family members
• I want to know what my child does when he/she plays outside
• My child needs permission, when he/she goes away after school
• My child needs to explain what he/she has done after school
(3) Psychological control
• I make my child feel guilty when he/she gets a low grade in school
• I make my child’s life difficult when he/she gets a low grade in school
• I act cold and unfriendly when my child behaves in a way I don’t like
• I don’t want to share time with my child when my child behaves in a way I don’t like
• I tell my child not to argue with adults
• I tell my child it’s better to admit during a quarrel with adults
• My child may not discuss his/her own ideas
• My general response to my child’s arguments is: “You will know better, once you are older”

## References

[CR1] Altenburg TM, Chinapaw MJ, van der Knaap ET, Brug J, Manios Y, Singh AS (2013). Longer sleep—slimmer kids: the energy-project. PLoS One.

[CR2] Aranceta J, Moreno B, Moya M, Anadón A (2009). Prevention of overweight and obesity from a public health perspective. Nutr Rev.

[CR3] Bawazeer NM, Al-Daghri NM, Valsamakis G, Ka A-R, Sabico SL, Huang TT, Mastorakos GP, Kumar S (2009). Sleep duration and quality associated with obesity among Arab children. Obesity.

[CR4] Beccuti G, Pannain S (2011). Sleep and obesity. Curr Opin Clin Nutr Marab Care.

[CR5] Berghöfer A, Pischon T, Reinhold T, Apovian CM, Sharma AM, Willich SN (2008). Obesity prevalence from a European perspective: a systematic review. BMC Public Health.

[CR6] Beyers W, Goossens L (1999). Emotional autonomy, psychosocial adjustment and parenting: interactions, moderating and mediating effects. J Adolesc.

[CR7] Boone-Heinonen J, Gordon-Larsen P, Adair LS (2008). Obesogenic clusters: multidimensional adolescent obesity-related behaviours in the US. Ann Behav Med.

[CR8] Brennan LK, Brownson RC, Orleans CT (2014). Childhood obesity policy research and practice: evidence for policy and environmental strategies. Am J Prev Med.

[CR9] CBS, Statistics Netherlands (2000). Standaarddefinitie allochtonen.

[CR10] Davison KK, Birch LL (2001). Childhood overweight: a contextual model and recommendations for future research. Obes Rev.

[CR11] de Jong E, Stocks T, Visscher TL, HiraSing RA, Seidell JC, Renders CM (2012). Association between sleep duration and overweight. Int J Obes.

[CR12] Delavari M, Sønderlund AL, Swinburn B, Mellor D, Renzaho A (2013). Acculturation and obesity among migrant populations in high income countries—a systematic review. BMC Public Health.

[CR13] Hasler G, Buysse DJ, Klaghofer R, Gamma A, Ajdacic V, Eich D, Rössler W, Angst J (2004). The association between short sleep duration and obesity in young adults: a 13-year prospective study. Sleep.

[CR14] Knutson KL (2012). Does inadequate sleep play a role on vulnerability to obesity?. Am J Hum Biol.

[CR15] Kocken PL, Schönbeck Y, Hemmeman L, Janssens ACJW, Detmar SB (2012) Ethnic differences and parental beliefs are important for overweight prevention and management in children; a cross-sectional study in The Netherlands. BMC Public Health 12(876):1–10 10.1186/1471-2458-12-867 PMC3508795 23057582

[CR16] Labree LJW, van de Mheen H, Rutten FFH, Foets M (2011). Differences in overweight and obesity among children from migrant and native origin: a systematic review of the European literature. Obes Rev.

[CR17] Lamborn SD, Mounts NS, Steinberg L, Dornbusch SM (1991). Patterns of competence and adjustment among adolescents from authoritative, authoritarian, indulgent, and neglectful families. Child Dev.

[CR18] Liu J, Zhang A, Li L (2012). Sleep duration and overweight/obesity in children: review and implications for pediatric nursing. J Spec Pediatr Nurs.

[CR19] Magee CA, Huang XF, Iverson DC, Caputi P (2010) Examining the pathways linking chronic sleep restriction to obesity. J Obes E1-E8 10.1155/2010/821710 PMC2925323 20798899

[CR20] Méjean C, Traissac P, Eymard-Duvernay S, Delpeuch F, Maire B (2009). Influence of acculturation among Tunisian migrants in France and their past/present exposure to the home country on diet and physical activity. Public Health Nutr.

[CR21] Moreno LA, Bel-Serrar S, Santaliestra-Pasías AM, Rodriguez G (2013). Obesity prevention in children. World Rev Nutr Diet.

[CR22] Murrin CM, Kelly GE, Tremblay RE, Kelleher CC (2012). Body mass index and height over three generations: evidence from the lifeways cross-generational cohort study. BMC Public Health.

[CR23] Nguyen DM, El-Serag HB (2010). The epidemiology of obesity. Gastroenterol Clin N Am.

[CR24] O’Dea JA, Dibley MJ, Rankin NM (2012). Low sleep and low socioeconomic status predict high body mass index: a 4-year longitudinal study of Australian schoolchildren. Pediatr Obes.

[CR25] Reilly JJ, Kelly J (2005). Long-term impact of overweight and obesity in childhood and adolescence on morbidity and premature mortality in adulthood: systematic review. Int J Obes.

[CR26] Reilly J, Bonatki M, Leary SD, Wells JC, Davey-Smith G, Emmett P, Steer C, Ness AR, Sherriff A (2011). Progression from childhood overweight to adolescent obesity in a large contemporary cohort. Int J Pediatr Obes.

[CR27] Rutters F, Gerver WJ, Nieuwenhuizen AG, Verhoef SPM, Westerterp-Plantenga MS (2010). Sleep duration and body-weight development during puberty in a Dutch children cohort. Int J Obes.

[CR28] Spiegel K, Tasali E, Penev P, van Cauter E (2004). Brief communication: sleep curtailment in healthy young men is associated with decreased leptin levels, elevated ghrelin levels, and increased hunger and appetite. Ann Intern Med.

[CR29] Steinberg L, Elmen JD, Mounts NS (1989). Authoritative parenting, psychosocial maturity, and academic success among adolescents. Child Dev.

[CR30] Stronks K, Kulu-Glasgow I, Agyemang C (2009). The utility of 'country of birth' for the classification of ethnic groups in health research: the Dutch experience. Ethn Health.

[CR31] Taheri S (2006). The link between short sleep duration and obesity: we should recommend more sleep to prevent obesity. Arch Dis Child.

[CR32] Van Cauter E, Knutson KL (2008). Sleep and the epidemic of obesity in children and adolescents. Eur J Endocrinol.

[CR33] Wong WW, Oriz CL, Lathan D, Moore NA, Konzelmann KL, Adolph AL, Smith EO, Butte NF (2013). Sleep duration of underserved minority children in a cross-sectional study. BMC Public Health.

